# Tongue Stiffness as Presentation of Antiphospholipid Syndrome: A Case Report

**DOI:** 10.7759/cureus.8584

**Published:** 2020-06-12

**Authors:** Hana Rajevac, Kelly Steed

**Affiliations:** 1 Internal Medicine, Icahn School of Medicine at Mount Sinai, Bronx, USA; 2 Rheumatology, James J. Peters VA Medical Center, Bronx, USA

**Keywords:** tongue stiffness, antiphospholipid syndrome, antiphospolipid antibodies, white matter changes on mri

## Abstract

Antiphospholipid syndrome (APS) is a systemic autoimmune disorder with marked thrombotic and inflammatory features driven by the presence of antiphospholipid antibodies (APLA). Here, we report a case of APS with a rare, atypical manifestation and discuss a differential diagnosis.

A 53-year-old male without significant past medical history presented with new onset of episodic tongue stiffness and dysarthria which lasted for about a minute over a period of three months. This was associated with intermittent right retro-orbital sharp pain radiating to the parietal area. He also reported swelling and stiffness of the third and fourth right proximal interphalangeal (PIP) joints lasting throughout the day. A physical exam revealed tongue fasciculations. As the MRI showed patchy white matter hyperintensities neurology initially suspected multiple sclerosis. However, cerebrospinal fluid (CSF) analysis including neuromyelitis optica (NMO) antibodies and oligoclonal antibodies was negative. Rheumatological work up was remarkable for positive antinuclear antibodies (ANA); anticardiolipin antibodies and lupus anticoagulant were positive 12 weeks apart. This, alongside with stable white matter changes on imaging was suspicious for an extra-criteria manifestation of antiphospholipid antibody syndrome.

The most commonly described neurological manifestations of APS are headache, transient ischemic attack (TIA), and stroke. Tongue stiffness as an initial symptom is quite unusual and, to the best of our knowledge has not been reported in medical literature. In patients with isolated neurological findings of unclear etiology, an autoimmune disease such as APS should be considered, and appropriate diagnostic work up should not be postponed. Unfortunately, positive laboratory markers can have a wide differential diagnostic panel. In addition, APS may mimic many diseases both in clinical presentation and MRI findings thus making the correct diagnosis challenging. However, studies show that, unlike multiple sclerosis (MS), white matter changes in APS remain static during the course of the disease. Identification of atypical presentations of APS is critical as prompt and correct medical management can improve patients’ quality of life and clinical outcomes.

## Introduction

Antiphospholipid syndrome (APS), also known as Hughes syndrome, is an autoimmune disease that can cause arterial, venous, or small-vessel thrombosis. A characteristic feature in women is pregnancy loss, defined as fetal death after 10 weeks, premature death due to severe preeclampsia or placental insufficiency, or multiple embryonic losses before 10 weeks. The main laboratory feature of APS is the presence of antiphospholipid antibodies (APLA) such as anticardiolipin, Lupus anticoagulant, anti-beta 2 glycoprotein I, and have been shown to enhance activation of platelets, endothelial cells and monocytes, thus causing an overproduction of tissue factor and thromboxane A2, as well an excessive activation of the complement factors. Inappropriate initiation of this pro inflammatory and prothrombotic cascade can result in diffuse thrombosis of and/or well-defined obstetrical manifestations [1-3]. 

Clinical manifestations of APS can be highly variable, but most commonly include deep vein thrombosis, pulmonary embolism, peripheral ischemia, livedo reticularis or neurological abnormalities such as transient ischemic attack (TIA) or stroke. Appropriate laboratorial work up with positive APLA, namely anticardiolipin antibodies (aCL), anti-beta 2 glycoprotein I (anti-b2GPI), or lupus anticoagulant (LA) antibodies would confirm the diagnosis [3-4]. Here, we report the case of a patient who presented with intermittent tongue stiffness for three months, associated with right retro-orbital and parietal area pain. The purpose of this report is to raise awareness for rare extra-criteria presentations of APS, and discuss a differential diagnosis.

## Case presentation

A 53-year-old African American male with past medical history of anxiety, dry eyes, lumbosacral radiculopathy, left rotator cuff tear, and osteoarthritis initially presented to his primary care physician complaining of sudden onset of episodic tongue stiffness and weakness, manifesting as episodes of slurred speech lasting for about a minute. Upon further questioning, he endorsed that these episodes were bothering him for the past three months, unrelated to the time of day or social events. These episodes were occasionally associated with intermittent sharp pain in the right retro-orbital area, radiating to the right parietal area described as tension-like sensation lasting for about eight hours. He denied any unintentional weight loss, did not experience any associated nausea, vomiting, photo, or phonophobia. Also he denied any focal weakness, numbness, and head trauma. The primary care physician recommended evaluation by a neurologist.

At the neurology office his exam was notable for tongue fasciculations which prompted further investigation modalities. 

He was subsequently referred to the rheumatology department. During the visit he reported episodes of slurred speech occurring two to three times a week, and right-sided tension type headaches. Additionally, he reported swelling and stiffness of the third and fourth right proximal interphalangeal (PIP) joints lasting throughout the day associating them with previous injuries. The remainder of review of system negative for scalp tenderness, polymyalgia rheumatica symptoms, fatigue, diplopia, vision changes or eye inflammation, lymphadenopathy, Raynaud's, oral ulcers or nasal ulcers, seizures, rash, or shortness of breath. There were also no fevers, no abdominal pain, no hematuria, and no personal history of blood clots.

Home medications included cholecalciferol, loratidine, gabapentin, duloxetine, and zolpidem tartrate. He endorsed a previous smoking history of five cigarettes a day for 20 years, denied illicit drugs or alcohol abuse, history of sexually transmitted infections, or any work-related or travel-related exposures. His family history was remarkable for fatal myocardial infarctions in two uncles who died at ages of 49 and 50, and coronary artery disease (CAD) in his mother with coronary artery stents placed in her 70s.

Vital signs afebrile, blood pressure 123/87 mmHg , pulse rate 77/min, respiration 20/min (Table 1). Physical exam was notable for tenderness to palpation of the swollen right third and fourth PIP joints, pea-sized mobile cervical lymph node on the left side. His lungs were clear to auscultation and his abdomen was not distended. There were no oral or nasal ulcers. His sensation in the facial and extremity dermatomes was preserved bilaterally. His strength in all extremities, including extension of the wrists and foot dorsiflexion was maintained. Motor strength 4+/5 over the right hip flexor. The rest of his motor strength exam 5/5 throughout.

**Table 1:** Vital signs of the patient on initial exam.

Vital signs	Value
Blood pressure	123/87 mmHg
Temperature	97.2 F
Pulse rate	77/min
Respiration	20/min

Laboratory tests revealed white blood cell (WBC) count of 7.3 k/cmm, hemoglobin 13.2 g/dL, platelet count of 378 k/cmm, comprehensive metabolic panel values within normal range. Erythrocyte sedimentation rate (ESR) normal, C-reactive protein (CRP) 11.3, complement within normal range, creatine phosphokinase (CPK) 223 (Table 2).

**Table 2:** Laboratory values of the patient during the initial encounter.

Laboratory test	Values
White blood cell count	7.3 k/cmm
Hemoglobin	13.2 g/dL
Platelet count	378 k/cmm
Comprehensive metabolic panel	All values within normal range
Erythrocyte sedimentation rate	12 mm/h
C-reactive protein	11.3 mg/L
Complement C3	134 mg/dL
Complement C4	41 mg/dL
Creatine phosphokinase	223 U/L

Infectious work up including gonorrhea, chlamydia, syphilis, lyme antigen was negative and HIV test nonreactive. Hepatitis B surface antibody positive, Hepatitis B surface antigen, and Hepatitis B core antibody were negative. Hepatitis C antibody was negative (Table 3).

**Table 3:** Infectious workup of the patient at 12 week interval.

Infectious workup	Result	Result 12 weeks later
Gonorrhea	Negative	Negative
Chlamydia	Negative	Negative
Syphilis	Negative	Negative
Lyme antigen	Negative	Negative
HIV AB & PCR	Nonreactive	Nonreactive
Hepatitis B surface antibody	Positive	Positive
Hepatitis B surface antigen	Negative	Negative
Hepatitis B core antibody	Negative	Negative
Hepatitis C antibody	Negative	Negative
Quantiferon TB Gold	Negative	Negative

Rheumatoid factor, ribonucleoprotein/Smith (RNP SM) antibody, antidouble strain (DS) DNA, and extractable nuclear antigen (ENA) panel were negative (Table 4).

**Table 4:** Rheumatological workup of the patient during the initial encounter.

Rheumatological workup	Result
Rheumatoid factor	Negative
Ribonucleoprotein/Smith (RNP SM) antibody	Negative
Anti double strain (DS) DNA	Negative
Extractable nuclear antigen (ENA) panel	Negative

Antinuclear antibody (ANA) was positive 1:160 with homogeneous pattern and 1:80 with nuclear pattern. Anticardiolipin antibody (aCL) IgM>150 12 weeks apart. ACL IgG and IgA <9. LA was also positive to 1.39 and 1.50 12 weeks apart (Table 5).

**Table 5:** Rheumatological work up of the patient during the initial encounter and 12 week follow up.

Rheumatological workup continued	Value initial	Value at 12 weeks
Antinuclear antibody (ANA) screen	Positive	N/A
ANA homogenous pattern	1:160	N/A
ANA nucleolar pattern	1:80	N/A
Lupus anticoagulant (LA)	1.39 ratio	1.50 ratio
Anticardiolipin antibody (aCL) IgM	>150 MPL U/mL	>150 MPL U/mL
aCL IgA	<9 APL U/mL	<9 APL U/mL
aCL IgG	<9 GPL U/mL	<9 GPL U/mL
Anti-β2glycoprotein-1 antibodies	<9 GPI units	<9 GPI units

Neuromyelitis optica (NMO) antibody in serum <1.5 U/mL. Cerebrospinal fluid (CSF) analysis: lyme IgG and IgM antibodies negative. Zero oligoclonal bands were observed in the CSF. CSF protein within normal limits 39.9, CSF glucose 75 mg/dL, angiotensin-converting enzyme (ACE) in CSF 1.1 U/L. CSF Gram stain negative and cultures negative (Table 6).

**Table 6:** Neurological work up of the patient.

Neurological work up	Value
Neuromyelitis optica (NMO) antibody serum	<1.5 U/mL
Lyme IgG and IgM Ab cerebrospinal fluid (CSF) analysis	Negative
Oligoclonal bands CSF analysis	0 (zero)
Protein CSF analysis	39.9 mg/dL
Glucose CSF analysis	75 mg/dL
Angiotensin-converting enzyme (ACE) CSF analysis	1.1 U/L
Gram stain/culture CSF analysis	Negative

Ultrasound (US) of the hands, done for the report of swelling and stiffness of the third and fourth right PIP joints, did not show any signs of synovitis, collections, or malformations.

**Figure 1:**
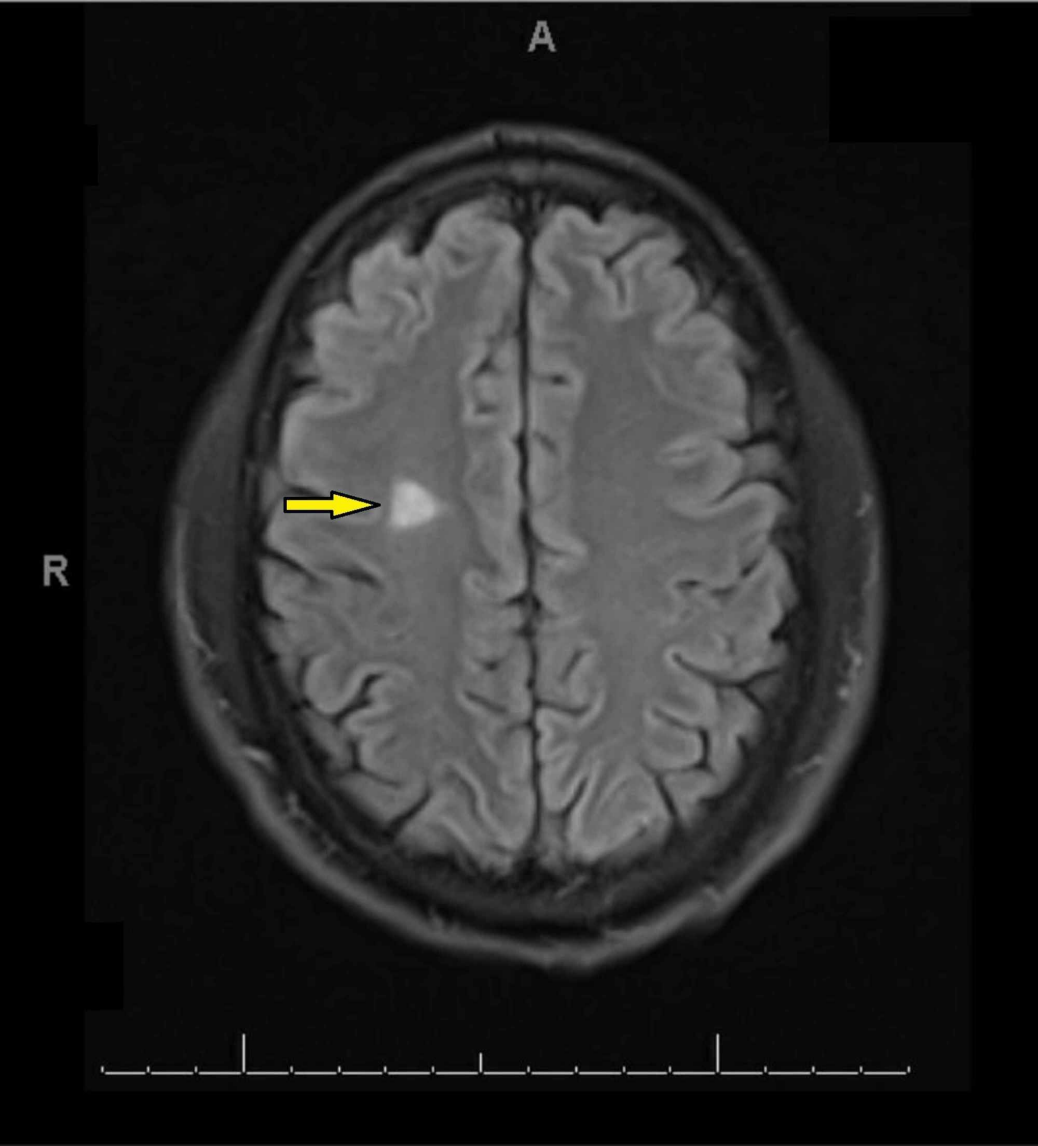
FLAIR/T2 weighted hyperintense signal foci in the right periventricular white matter on MRI.

**Figure 2:**
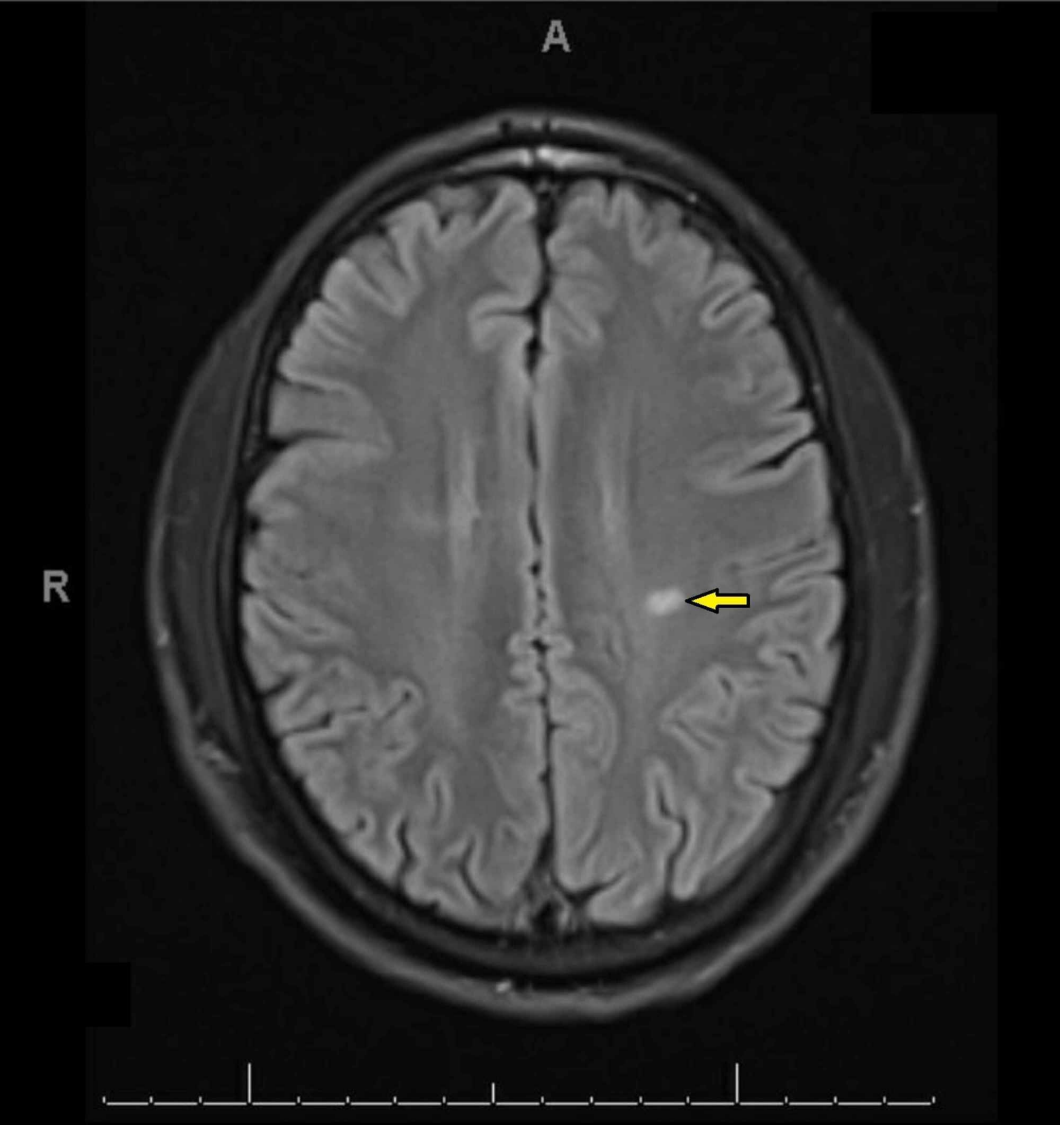
FLAIR/T2 weighted hyperintense signal foci in the left periventricular white matter on MRI.

**Figure 3:**
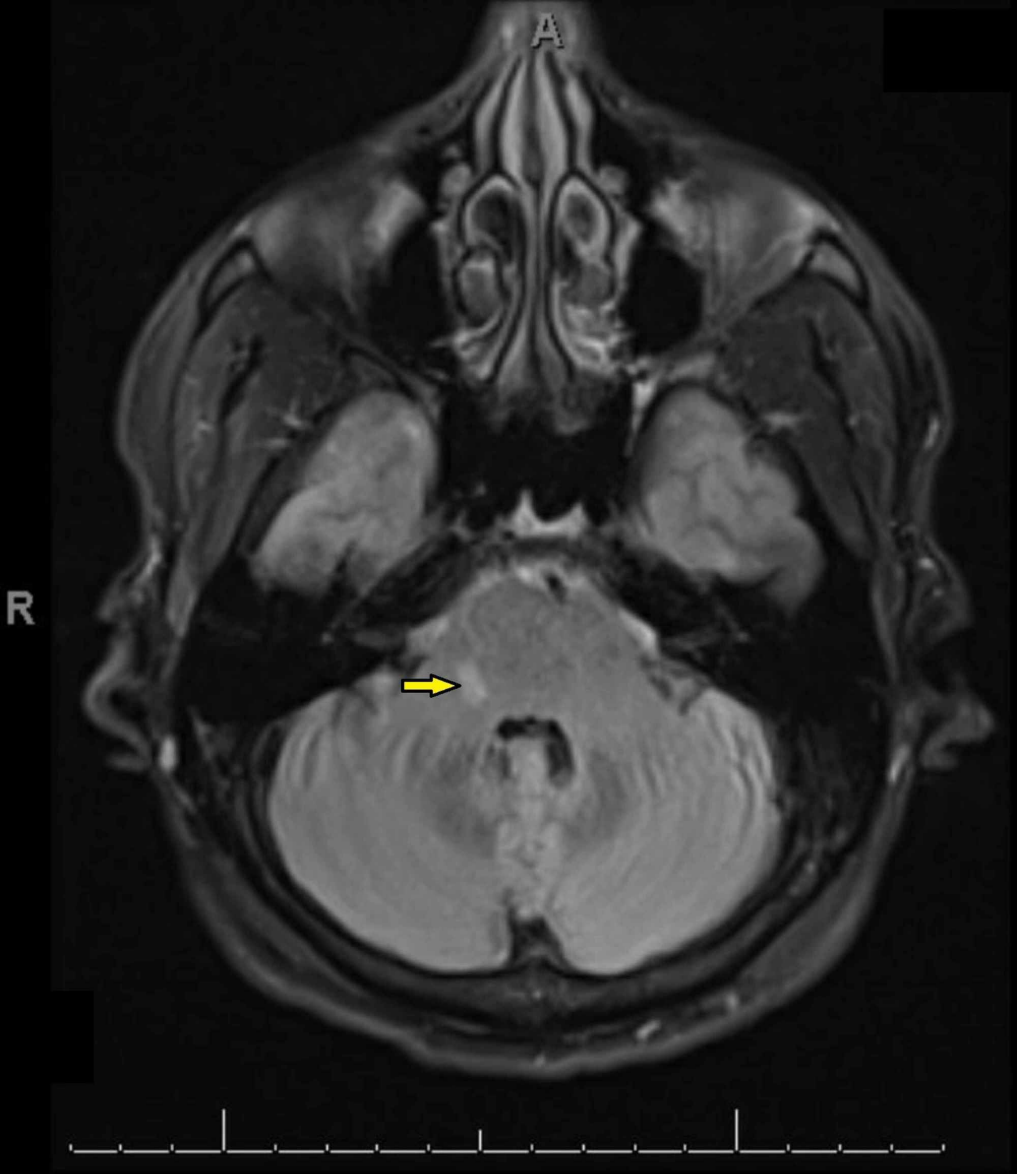
FLAIR/T2 weighted hyperintense signal foci in the right brachium pontis on MRI.

MRI of the brain (with/without contrast) showed several scattered FLAIR/T2 weighted hyperintense signal foci in bilateral periventricular white matter perpendicular to the ventricular system, and hyperintensity also noted in the right brachium pontis. Although the corpus callosum appeared to be intact the neuroradiologist could not exclude a possible demyelinating process (Figures 1-3). Magnetic resonance angiogram (MRA) of the brain was negative for occlusion/vasculitis.

Once after latent and ongoing infectious processes were ruled out, the patient was offered treatment with rituximab in accordance with the recent APL RITAPS trial [5], which reviewed the role of rituxan in APS with noncriteria manifestations.

The patient deferred rituximab and was placed on daily aspirin 81 mg PO for primary prophylaxis. The patient has agreed for the close follow up. The patient’s ANA and aCL remained elevated. Repeated MRI of the brain four months and 16 months after the initial imaging has shown no progression of the white matter changes. Aspirin was continued with the agreement of the patient. The patient is still experiencing the episodes of the tongue stiffness, although less frequently. The tongue stiffness/dysarthria are occurring independently of the headache which is now generalized.

## Discussion

Here we report a case of atypical APS which manifested with three months of intermittent tongue stiffness, initially assumed to represent either isolated cranial nerve palsy or an early manifestation of multiple sclerosis until the diagnosis of APS was finally suspected.

The APLA can be detected in a variety of rheumatological diseases as well as in case of malignancies and infections. In some instances, ALPA have be associated with use of specific medications.

Malignancies which can be associated with APLA include solid tumors of the lung, colon, cervix, ovary, breast, prostate, and bone; Hodgkin disease and non-Hodgkin lymphoma; myeloid and lymphocytic leukemias, primary myelofibrosis, or polycythemia vera [6]. APLA can be transiently detected in patients with different types of infections, and they can lead to thrombotic events, albeit rarely. Bacterial infections such as lyme disease (borreliosis), tuberculosis, leprosy, leptospirosis, syphilis, infective endocarditis, and Klebsiella infections can cause elevation in APLA. Similarly viral infections including Hepatitis A, B, and C, mumps, HIV, parvovirus, cytomegalovirus, varicella-zoster, Epstein-Barr virus (EBV), rubella, as well as parasitic infections like malaria and pneumocystis jiroveci have been associated with APLA. Medications known to potentially cause a transient, heterogenous ALPA profile are propranolol, hydralazine, procainamide, quinidine, quinine, alpha interferon, amoxicillin, chlorothiazide, oral contraceptives, chlorpromazine, and phenytoin [7].

The APLA are also found in primary (or isolated) APS or associated with other autoimmune entities.

Primary APS occurs in the absence of any other related disease. In rare cases (about 1% of isolated APS), the APS manifests as a rapid organ failure due to microthrombi formation in multiple organs and systemic inflammatory response; this is termed "catastrophic antiphospholipid syndrome" (CAPS or Asherson's syndrome) and is associated with a high risk of death.

Importantly, significant APLA levels have been detected in up to approximately 30%-40% of patients with systemic lupus erythematosus (SLE) [8-9]. SLE patients with APLA in the blood appear more likely to develop thrombosis and/or experience obstetric complications such as miscarriages. Overall, the presence of APLA is associated with worse SLE disease course due to a higher incidence of irreversible organ damage and an increased mortality rate. The presence or absence of concomitant SLE may impact the clinical or serological expression of APS. APS individuals with associated SLE are more frequently presented with arthritis, epilepsy, glomerular thrombosis, myocardial infarction, autoimmune hemolytic anemia, and livedo reticularis [9].

A subset of APLA has also been found in patients who have many other rheumatic diseases like psoriatic arthritis, rheumatoid arthritis, Sjögren syndrome, systemic sclerosis, or poststreptococcal rheumatic fever.

Revised Saporo criteria for the diagnosis of APS require at least one laboratory criterion and one clinical criterion. The laboratory criteria are represented by the presence of a medium or high titer of APLA detected by ELISA testing in two or more samples obtained at least 12 weeks apart. APLA comprise: anticardiolipin (aCL), LA, anti-beta 2 glycoprotein I ( anti-b2GPI) antibodies. As per the clinical criteria, this is met in case of one or more episodes of arterial, venous or small vessel thrombosis in any tissue, pregnancy morbidity manifested as unexplained perinatal deaths, premature births, or history of one or more miscarriages. Thrombotic events such as stroke, myocardial infarction, and pulmonary embolism are the most common causes of death in APS.

The annual rate of a first vascular event described in the literature was 0.65% in single positivity APLA carriers, but it increased twofold in carriers of double or triple APLA positivity (1.27%) [10-11]. Pengo et al. suggest in their study an annual rate of first thrombotic event up to 5.3%, in a cohort of 104 triple APLA positive patients with no previous history of thrombosis [11].

Symptoms such as tongue stiffness and parietal headache can be attributed to more common diseases such as palsies of various cranial nerves, atypical migraine attacks, multiple sclerosis (MS), stroke, or TIA. Isolated tongue stiffness in the absence of headache has also been described in case of meningioma of foramen magnum [12].

Imaging findings of T2 weighted hyper intense foci in the periventricular white matter bilaterally can be associated with Alzheimer disease, small vessel disease, stroke or with a demyelinating process such as MS [13]. However, the patient did not have any other neurological defects. The absence of oligoclonal antibodies and the lack of disease progression on the repeat MRI study is less consistent with a primary neurological disorder. In addition, the presence of persistently positive LA and aCL antibodies, on two separate occasions at least 12 weeks apart, are highly suggestive of a rheumatological disorder.

The most common neurological manifestations of APS described in the literature are headaches, TIA, and stroke [14-15]. Isolated tongue stiffness is quite an unusual symptom. In patients with isolated neurological findings of unclear etiology, an autoimmune disease such as atypical APS should be considered as a differential diagnosis. Appropriate rheumatological workup should not be postponed. A neurological APS may mimic MS in clinical presentation, and even give similar findings on an MRI [16]. Demyelinating white matter lesions involving the periventricular area and/or both cerebral lobes are characteristic MRI findings in patients who suffer from APS [17-18].

The absence of oligoclonal bands in the CSF analysis could be a clue in favor of a neurological presentation of APS, although it does not fully rule out MS. In this particular case, white matter changes had not shown progression on MRI during the follow up period. Previous literature seems to favor a diagnosis of APS when periventricular white matter lesions remain unchanged over time [19-20]. These nonspecific manifestations of APS can easily be overlooked, if the appropriate additional diagnostic tests are not conducted.

This case report sought to highlight a subtle and quite unusual presentation of APS and encourage clinicians to obtain differential diagnostic work up when facing atypical symptoms.

## Conclusions

Tongue stiffness can be the initial symptom of an autoimmune disease. Extra-criteria APS should be considered in patients who only have either laboratory or clinical criteria for APS, but not both as in typical presentations. Abnormalities on brain imaging need to be interpreted with caution and might require interval reassessment in order to make the correct diagnosis. Once a suspicion of APS is present appropriate diagnostic work up and medical management are critical to improve long-term outcomes and quality of life.
